# Classification of Computed Tomography Images in Different Slice Positions Using Deep Learning

**DOI:** 10.1155/2018/1753480

**Published:** 2018-07-16

**Authors:** Hiroyuki Sugimori

**Affiliations:** Faculty of Health Sciences, Hokkaido University, Sapporo 060-0812, Japan

## Abstract

This study aimed at elucidating the relationship between the number of computed tomography (CT) images, including data concerning the accuracy of models and contrast enhancement for classifying the images. We enrolled 1539 patients who underwent contrast or noncontrast CT imaging, followed by dividing the CT imaging dataset for creating classification models into 10 classes for brain, neck, chest, abdomen, and pelvis with contrast-enhanced and plain imaging. The number of images prepared in each class were 100, 500, 1000, 2000, 3000, 4000, 5000, 6000, 7000, 8000, 9000, and 10,000. Accordingly, the names of datasets were defined as 0.1K, 0.5K, 1K, 2K, 3K, 4K, 5K, 6K, 7K, 8K, 9K, and 10K, respectively. We subsequently created and evaluated the models and compared the convolutional neural network (CNN) architecture between AlexNet and GoogLeNet. The time required for training models of AlexNet was lesser than that for GoogLeNet. The best overall accuracy for the classification of 10 classes was 0.721 with the 10K dataset of GoogLeNet. Furthermore, the best overall accuracy for the classification of the slice position without contrast media was 0.862 with the 2K dataset of AlexNet.

## 1. Introduction

In the field of computer vision, deep learning with a convolutional neural network (CNN) [1] can be used to attain precise general image classification. Recently, deep learning has been increasingly used in medical imaging [2–17]. Arguably, deep learning has several potential abilities, including object detection [9, 10] and image segmentation. Typically, medical images differ from general images in that medical images only depict human structures with no background structure other than that of a human body. Previously, some studies have reported the classification of the scan position with computed tomography (CT) imaging using deep learning [18, 19]; however, it evaluated the accuracy of classification only when detecting scan slice positions. Thus, research has recognized the necessity of the enhancement information using contrast media for tumor diagnosis [20, 21] because radiologists commonly refer to the slice position, organ structure status, and presence of an organ or tumor enhancement. Further, information concerning the position and presence of contrast media is one of the critical factors for the basic requirement for automatic diagnosis using deep learning. Fundamentally, deep learning requires several images [22] to create classification models, although human structures comprise variable organ structures with different sizes in each subject. Moreover, related works with deep learning in CT images have recently been reported dealing with detections of anatomies and tumors [23–26]. In these techniques, whole body CT images could not be used for deep learning because those images have to be classified as concerning region in advance. However, till date, no study has reported how many images are required for the classification of CT images, including contrast enhancement data. If the precise classification of CT images has done as a preprocessing, the automatic diagnosis using deep learning for whole body images will be more practical technique. Thus, this study aimed at elucidating the relationship between the number of CT images, including data concerning the accuracy of models and contrast enhancement for creating classification models.

## 2. Materials and Methods

### 2.1. Subjects

Totally, 1539 patients (males, 815; females, 724; mean age ± standard deviation (SD), 59.9 ± 18.5 years) who underwent contrast or noncontrast CT imaging of the brain, neck, chest, abdomen, or pelvis in January 2016 were included in the study. The study protocol was approved by the Ethics Committee of the Hokkaido University Hospital (Sapporo, Japan).

### 2.2. Datasets

The dataset of CT images for creating classification models was divided into 10 classes for the brain, neck, chest, abdomen, and pelvis with contrast-enhanced (CE) and non-contrast-enhanced CT, which was defined as plain (P). The number of images prepared for each class were 100, 500, 1000, 2000, 3000, 4000, 5000, 6000, 7000, 8000, 9000, and 10,000; the datasets were named 0.1K, 0.5K, 1K, 2K, 3K, 4K, 5K, 6K, 7K, 8K, 9K, and 10K. We used these images of 90% of the data for training and 10% for validation for creating classification models. In addition, 1000 images from each class other than the datasets mentioned above were prepared for testing models.  Table 1 presents the name of all such datasets with complete details.

**Figure 1:**
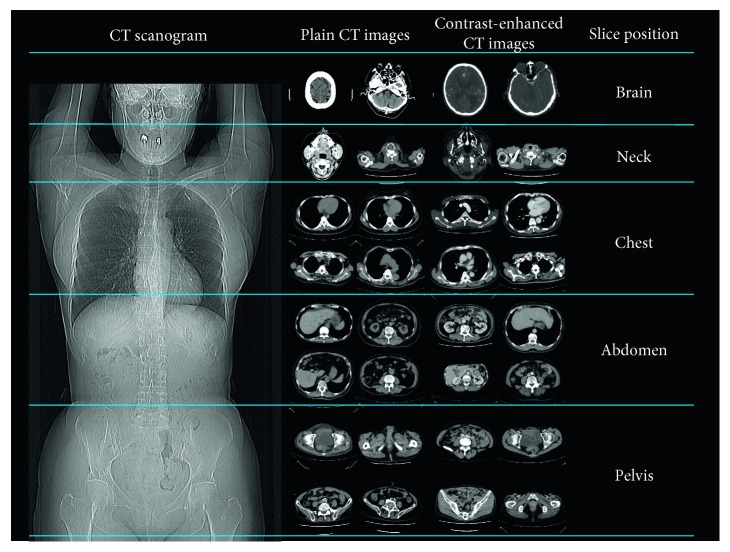
Range of the slice location and example image of 10 classes: the brain, neck, chest, abdomen, and pelvis.

The image ranges of each class were defined as follows: brain, slice from the anterior tip of the parietal bone to the foramen magnum; neck, slice from the foramen magnum to the pulmonary apex; chest, slice from the pulmonary apex to the diaphragm; abdomen, slice from the diaphragm to the top of an iliac crest; and pelvis, slice from the top of an iliac crest to the distal end of the ischium.  Figure 1 indicates the image ranges for each class.

Furthermore, CE was defined as the state of intravascular injection of contrast media in the examination. We did not consider the timing of scans from the injection. Criteria for exclusion from the dataset were CT images with excessive magnification, the reconstruction kernel of the bone or lung, nothing (above the anterior tip of the parietal bone), and arms or legs only.

### 2.3. Preprocessing for Creating Models

We retrieved CT images from the Picture Archiving and Communication System. Next, to convert images for the training database, we converted these CT images from the digital imaging and communications in medicine (DICOM) to the joint photographic experts group (JPEG) file format using dedicated DICOM software (XTREK View; J-Mac System Inc., Sapporo, Japan). In addition, the window width and level of the DICOM image were used with preset values in the DICOM-tag. Next, we converted all JPEG images to grayscale 8-bit images sized 512 × 512 pixels, followed by sorting the converted JPEG files into particular folders according to image classes. Furthermore, we used the NVIDIA Deep Learning GPU Training System (NVIDIA DIGITS; NVIDIA Corporation, Santa Clara, CA), the conversion software of the training database, and an authoring software for deep learning. Finally, the database type was set to the lightning memory-mapped database (LMDB).

### 2.4. Training for Creating Models

**Figure 2:**
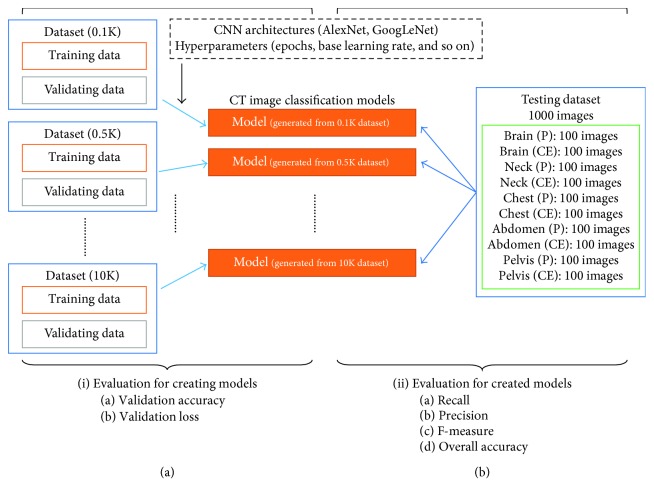
Schematic of the datasets, creating models, and validation of creating models. (a) Workflow of creating models and (b) workflow of the evaluation of created models. (i) Evaluation points for creating models and (ii) evaluation points for the created models.

Figure 2 outlines the training for creating models. We used the authoring software NVIDIA DIGITS for deep learning, a deep learning optimized machine with two GTX1080 Ti GPUs with 11.34 TFlops single precision, 484 GB/s memory bandwidth, and 11 GB memory per board. The convolutional architecture for fast feature embedding (Caffe) [27] constituted the deep learning framework, which was worked on the NVIDIA DIGITS. Here, we compared CNN architectures, which could be selected on the NVIDIA DIGITS, between the 16-layer AlexNet [28] and 22-layer GoogLeNet [29]. The training model hyperparameters were used as a default on the software (Table 2), and the maximum training epoch was set to 30. The initial learning rate was set at 0.01; it was later dropped by one-tenth following every 10 epochs of training.

In both CNN architectures, training was acquired three times for each dataset, followed by recording the best accuracy and loss of validation and calculating the mean value. We evaluated these results using datasets and CNN architectures, respectively. Moreover, the duration from the start of training to the complete creation of the model was assessed in each dataset.

### 2.5. Evaluation of Created Models

**Figure 3:**
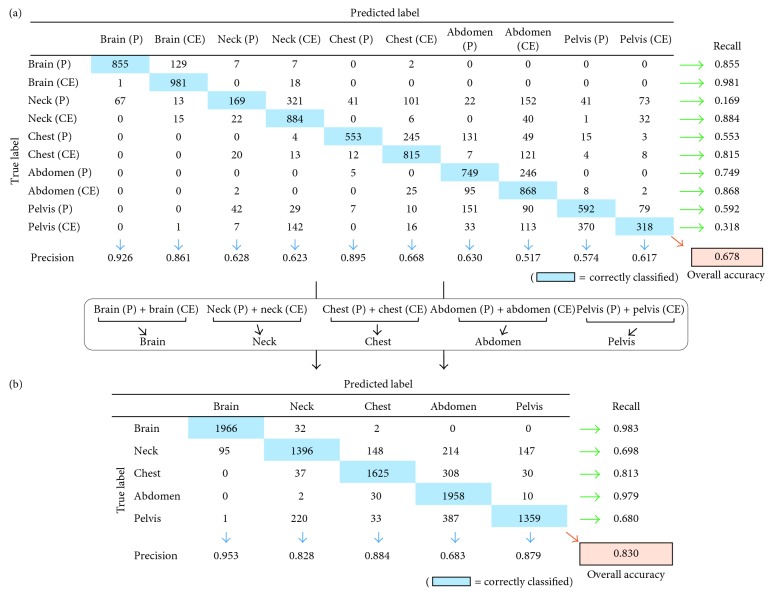
Computing different measures from the confusion matrix. The precision and recall of each class and the overall accuracy were calculated. (a) Confusion matrix with 10 classes and (b) confusion matrix of each slice location, calculated from (a).

Figure 2 outlines the training for the evaluation of created models. The confusion matrix obtained by an independent dataset was intuitively a fair indicator of the performance of the created models because training accuracy was the only result that was repeatedly evaluated using the same dataset.  Figure 3 shows examples of the confusion matrix. To evaluate the created models, we applied the dataset for the testing model (Section 2.2). For training with 10 classes, which presented as a 10 × 10 table, all performance measures were based on hundred numbers obtained by applying the classifier to the test dataset. Moreover, the confusion matrix comprised columns and rows corresponding to the predicted and the true image label, respectively. The same position was integrated for evaluating the positional detection ability, for example, brain (P) and brain (CE) as to the brain; the confusion matrix was created as a 5 × 5 table.

Here, the confusion matrix generated four parameters: precision, recall, F-measure, and overall accuracy. Precision was presented as a ratio of how many images were correctly predicted to produce the predicted labels; recall as a ratio presented how many of these were correctly classified. Furthermore, we defined F-measure as the harmonic mean of precision and recall:

(1) $$\begin{array}{c} \text{F}‐\text{measure}=\frac{\left(2\times \text{recall}\times \text{precision}\right)}{\left(\text{recall}+\mathrm{precision}\right)}\text{.} \end{array}$$

Overall accuracy was presented as the ratio of the number of correctly classified images in all test images. Furthermore, we evaluated the confusion matrix thrice based on the number of created models.

### 2.6. Statistical Analysis

Precision, recall, F-measure, and overall accuracy were presented as mean ± SD regardless of the dataset. In addition, the accuracy of validation for training, recall, precision, F-measure, and overall accuracy were evaluated at mean values higher than 0.80, 0.85, 0.90, or 0.95, and the loss of validation for training was evaluated at mean values less than 0.20, 0.15, 0.10, or 0.05. The time for training the model was evaluated as the mean time. The best overall accuracy was recorded from all datasets. Besides, the comparison of CNN architecture irrespective of the dataset was evaluated using the Mann–Whitney *U* test. Furthermore, we used the Steel–Dwass test for the multiple comparison of the slice position for the recall, precision, and F-measure and the comparison of the CNN architecture irrespective of the dataset. *P* < 0.05 was considered statistically significant.

## 3. Results and Discussion

### 3.1. Results

#### 3.1.1. Evaluation of Training of Models

Table 3 presents the accuracy of validation, loss of validation, and time taken for training the model. Datasets from 4K to 10K on AlexNet and 2K to 10K on GoogLeNet had an accuracy of validation of >0.95. In addition, datasets of 10K on AlexNet and from 5K to 10K, except for 6K, on GoogLeNet had loss of validation of <0.05. The mean accuracy of validation for AlexNet and GoogLeNet was 0.87 ± 0.19 and 0.90 ± 0.16, respectively, and a significant difference was observed between CNN architectures (*P*=0.0027). The mean loss of validation for AlexNet and GoogLeNet was 0.35 ± 0.52 and 0.27 ± 0.43, respectively, and a significant difference was observed between CNN architectures (*P*=0.0036). Furthermore, the mean time of training model for AlexNet and GoogLeNet was 16.4 ± 11.45 minutes and 43.42 ± 31.78 minutes, respectively, and a significant difference was observed between CNN architectures (*P*=0.0003).

#### 3.1.2. Evaluation of Created Models

*(1) Comparison of All 10 Classes.* Table 4 presents the recall, precision, and F-measure for each class for both AlexNet and GoogLeNet. The precision of the recall for brain (P) for AlexNet and GoogLeNet was >0.80; however, no parameters of the recall and F-measure were >0.80.  Table 5 presents the recall, precision, F-measure, and overall accuracy for each dataset of AlexNet and GoogLeNet. No parameters in all datasets were >0.80. The mean recall for AlexNet and GoogLeNet was 0.56 ± 0.28 and 0.58 ± 0.28, respectively, and no significant difference was observed between CNN architectures (*P*=0.4052). The mean precision for AlexNet and GoogLeNet was 0.58 ± 0.23 and 0.59 ± 0.22, respectively, and no significant difference was observed between CNN architectures (*P*=0.2496). The mean F-measure for AlexNet and GoogLeNet was 0.54 ± 0.24 and 0.56 ± 0.24, respectively, and no significant difference was observed between CNN architectures (*P*=0.1918). In addition, the mean overall accuracy for AlexNet and GoogLeNet was 0.56 ± 0.14 and 0.58 ± 0.16, respectively, and no significant difference was observed between CNN architectures (*P*=0.2601). Furthermore, the best overall accuracy for the classification of 10 classes was 0.721, which was obtained with dataset #3 of 10K of GoogLeNet.

**Figure 4:**
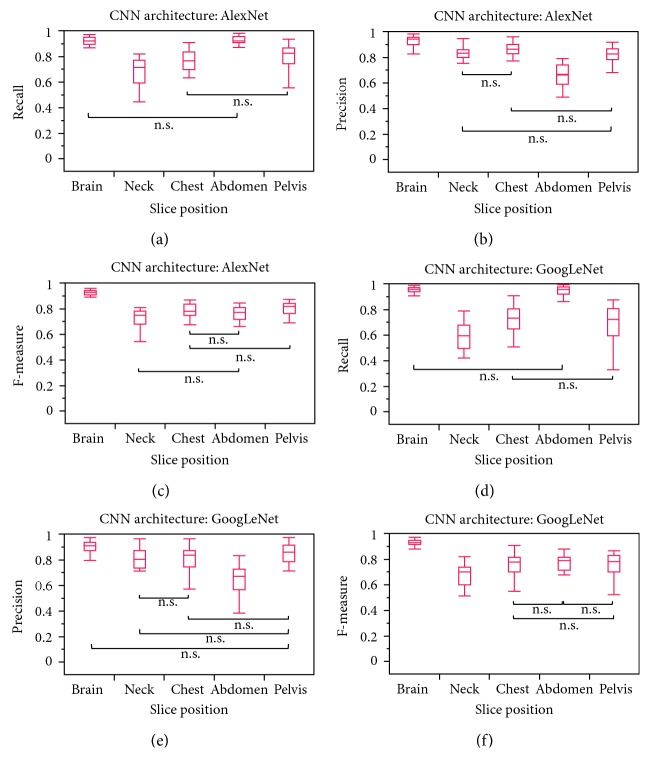
(a) Recall, (b) precision, and (c) F-measure for each slice position with AlexNet. (d) Recall, (e) precision, and (f) F-measure for each slice position with GoogLeNet (without specific marks indicates significant differences between each group; ^∗^n.s., no significant differences between the two groups, Steel–Dwass test).

*(2) Comparison of Each Slice Position.* Figure 4 indicates the recall, precision, and F-measure for each slice position for AlexNet and GoogLeNet. The mean recall for the brain in GoogLeNet and the abdomen in AlexNet and GoogLeNet was >0.90 and that for the brain in AlexNet was >0.85. The mean precision for the brain in AlexNet and GoogLeNet was >0.85 and that for the neck and chest in AlexNet and the pelvis in AlexNet and GoogLeNet was >0.80. In addition, the mean F-measure for the brain in AlexNet and GoogLeNet was >0.85.  Table 6 presents the recall, precision, F-measure, and overall accuracy for each dataset in AlexNet and GoogLeNet. Furthermore, the best overall accuracy for the classification of the slice position was 0.862, which was obtained with dataset #3 of 2K of AlexNet.

### 3.2. Discussion

In this study, a total of 12 datasets for the training models were compared for evaluating models. Regarding the training model, datasets with >4000 images/class on AlexNet and >2000 images/class on GoogLeNet could obtain an accuracy >0.95. Regarding validation loss, a higher number of images per slice were essential to reduce the loss. In addition, the training time in the CNN architecture of GoogLeNet was approximately double than that of AlexNet. The significant difference in training models between AlexNet and GoogLeNet occurred because the CNN structures of GoogLeNet had several layers in detail. Regarding the evaluation of created models with 10 classes, CT images of the brain (P) and brain (CE) exhibited higher recall and precision because the anatomy and size of the brain were different from that of the other parts of the body, indicating that the F-measure value was higher in the brain than in other organs. However, other classes of the recall and precision exhibited lower classes of the brain; in fact, the recall of the neck (P) was particularly lower than that of the others, perhaps, because the CT images of the neck were affected by the artifact from the metal of the artificial tooth. Reportedly, the recall of the classification could be lower because these artifacts from the tooth, which rendered differentiating the enhancement by contrast media difficult, affected images in the form of noise [30]. Typically, the difference between P and CE images could only be reflected in the vascular because of the few structures of the strong enhancement by contrast media in the neck. In addition, the lower recall of the neck and pelvis could be attributed to the similar symmetric structure of the iliac bone and scapula on the backside of the body. Precisely, the lower recall of the neck and pelvis was caused not only by the difference in the enhancement by contrast media but also the structure. The calcification of the iliac artery was one of the causes of confusion between P and CE images, because the CT value was higher at the calcification of the artery, which resembled the enhanced vascular. Reportedly, the calcification of the iliac artery, which is associated with some diseases [31, 32], was one of the causes of confusion between P and CE images, because the calcification of the artery was depicted like an enhanced vascular. Regarding the evaluation of the created models for each slice position, CT images of the brain exhibited higher recall and precision for the same reason as that for the 10 classes. As the recall of the abdomen was higher, it leaves no bone structure, except for the spine, in the range of the abdomen in this study. Of note, the lower recall of the neck and pelvis was formed by the similar symmetric structure because the contrast of the bone was primarily characterized as a feature of CT images. In addition, the features over the two regions were unclear because the borders between the neck and chest and between the chest and abdomen were overlapped in each position. In case of removing the overlapped range of images from the dataset, the recall and precision would probably be higher; this assumption was supported by the fact that the dataset of 1K had already exhibited an overall accuracy of approximately 0.8. Previously, some studies have reported the classification of CT images [18, 19]. However, the number of CT images in each class was one-tenth compared with the datasets in this study. Moreover, the test dataset for the validation of created models was 10,000 images of 1000 images per class; in this respect, this study differed from others. Regarding the CNN architecture, the time for the training model of AlexNet was faster than that for GoogLeNet, because the larger number of layers and complicated CNN structure of GoogLeNet affected the time for the training model. In addition, no significant differences existed between both of them regarding the evaluation of created models. Thus, AlexNet would be useful for being rapid and simple. This study has some limitations. First, we used the default CNN architecture and hyperparameters. Because the classification of general images with deep learning was sophisticated [28, 29], the major change in the CNN architecture for medical images might be unstable for the optimization of creating models. Second, the created models were evaluated using the hold-out method which was known as the simplest kind of cross validation. With regard to the cross validation, we focused on the simple tendency for the effect of the size of dataset though the k-fold cross validation has been reported [33]. The reason of choice for the hold-out method was that the small number of dataset had not enough the number of images because the k-fold method has to divide the data into k-equally sized folds. However, we could present the basic tendency for the size of the dataset in this study. Third, all images of the dataset in this study were original CT images. Thus, giving a variety to the feature of the dataset was necessary for this study, although the data augmentation [19] was critical for not only the increased number of images but also the repeated usage of similar images for the training. However, the number of patients in this study was approximately 1539 with >100,000 images; thus, we believe that the results of this study could serve as a reference for further investigation in the future.

## 4. Conclusions

This study elucidates the relationship between the number of CT images, including data about contrast enhancement for creating classification models, and accuracy of models. The time for training models of AlexNet was faster than that for GoogLeNet. Furthermore, the best overall accuracy for the classification of 10 classes was 0.721 with the dataset of 10K of GoogLeNet, and the best overall accuracy for the classification of the slice position regardless of contrast media was 0.862 with the dataset of 2K of AlexNet.

## Figures and Tables

**Table 1:** Names of datasets and the number of images in each label.

	Names of datasets												
	0.1K	0.5K	1K	2K	3K	4K	5K	6K	7K	8K	9K	10K	Testing dataset
Brain (P)	100	500	1000	2000	3000	4000	5000	6000	7000	8000	9000	10,000	100
Brain (CE)	100	500	1000	2000	3000	4000	5000	6000	7000	8000	9000	10,000	100
Neck (P)	100	500	1000	2000	3000	4000	5000	6000	7000	8000	9000	10,000	100
Neck (CE)	100	500	1000	2000	3000	4000	5000	6000	7000	8000	9000	10,000	100
Chest (P)	100	500	1000	2000	3000	4000	5000	6000	7000	8000	9000	10,000	100
Chest (CE)	100	500	1000	2000	3000	4000	5000	6000	7000	8000	9000	10,000	100
Abdomen (P)	100	500	1000	2000	3000	4000	5000	6000	7000	8000	9000	10,000	100
Abdomen (CE)	100	500	1000	2000	3000	4000	5000	6000	7000	8000	9000	10,000	100
Pelvis (P)	100	500	1000	2000	3000	4000	5000	6000	7000	8000	9000	10,000	100
Pelvis (CE)	100	500	1000	2000	3000	4000	5000	6000	7000	8000	9000	10,000	100
Total number of images	1000	5000	10,000	20,000	30,000	40,000	50,000	60,000	70,000	80,000	90,000	100,000	1000
For training	900	4500	9000	18,000	27,000	36,000	45,000	54,000	63,000	72,000	81,000	90,000	-
For validation	100	500	1000	2000	3000	4000	5000	6000	7000	8000	9000	10,000	-

**Table 2:** Hyperparameters of training models.

	CNN architecture	
	AlexNet	GoogLeNet
Training epochs	30	
Snapshot interval	10	
Validation interval	1	
Random seed	None	
Batch size of training	128	32
Batch size of validation	32	16
Solver type	Stochastic gradient descent (SGD)	
Base learning rate	0.01	
Policy	Step down	
Step size (%)	33	
Gamma	0.1	

**Table 3:** Accuracy and loss of validation for training models.

	CNN architecture	Dataset											
		0.1K	0.5K	1K	2K	3K	4K	5K	6K	7K	8K	9K	10K
Accuracy of validation	AlexNet	0.27	0.77	0.87	0.92	0.93	0.95	0.95	0.96	0.97	0.97	0.97	0.97
	GoogLeNet	0.40	0.78	0.93	0.96	0.96	0.97	0.98	0.97	0.97	0.98	0.98	0.98

Loss of validation	AlexNet	2.00	0.57	0.36	0.21	0.21	0.15	0.14	0.12	0.11	0.11	0.10	0.09
	GoogLeNet	1.59	0.55	0.24	0.12	0.12	0.10	0.09	0.10	0.09	0.08	0.08	0.07

Time for training (min)	AlexNet	0.8	2.1	3.8	7.3	10.7	14.2	17.4	21.3	24.7	27.9	31.8	34.8
	GoogLeNet	1.1	4.9	9.5	18.7	28.9	38.5	48.3	45.7	67.6	76.5	86.4	95.0

**Table 4:** Recall, precision, and F-measure for each class in AlexNet and GoogLeNet.

	Recall		Precision		F-measure	
	AlexNet	GoogLeNet	AlexNet	GoogLeNet	AlexNet	GoogLeNet
Brain (P)	0.71	0.77	0.81	0.80	0.74	0.78
Brain (CE)	0.77	0.79	0.72	0.70	0.73	0.73
Neck (P)	0.20	0.17	0.46	0.46	0.25	0.22
Neck (CE)	0.53	0.57	0.46	0.55	0.46	0.54
Chest (P)	0.44	0.49	0.68	0.67	0.52	0.55
Chest (CE)	0.71	0.67	0.63	0.62	0.65	0.64
Abdomen (P)	0.61	0.69	0.57	0.54	0.56	0.59
Abdomen (CE)	0.73	0.74	0.48	0.47	0.56	0.57
Pelvis (P)	0.52	0.52	0.48	0.53	0.48	0.51
Pelvis (CE)	0.42	0.40	0.52	0.60	0.44	0.47

**Table 5:** Recall, precision, F-measure, and overall accuracy for each dataset in AlexNet and GoogLeNet.

	CNN architecture	Dataset											
		0.1K	0.5K	1K	2K	3K	4K	5K	6K	7K	8K	9K	10K
Recall	AlexNet	0.20	0.39	0.52	0.53	0.61	0.67	0.61	0.67	0.67	0.65	0.64	0.62
	GoogLeNet	0.15	0.38	0.57	0.62	0.65	0.68	0.61	0.65	0.69	0.62	0.67	0.69

Precision	AlexNet	0.20	0.39	0.53	0.54	0.61	0.67	0.65	0.69	0.70	0.68	0.67	0.64
	GoogLeNet	0.15	0.41	0.58	0.62	0.65	0.68	0.63	0.66	0.71	0.64	0.70	0.70

F-measure	AlexNet	0.14	0.35	0.49	0.50	0.60	0.65	0.59	0.65	0.66	0.63	0.62	0.59
	GoogLeNet	0.11	0.35	0.54	0.61	0.64	0.67	0.59	0.63	0.67	0.60	0.65	0.68

Overall accuracy	AlexNet	0.20	0.39	0.52	0.53	0.61	0.66	0.61	0.67	0.67	0.65	0.63	0.62
	GoogLeNet	0.15	0.38	0.57	0.62	0.65	0.68	0.61	0.65	0.69	0.62	0.67	0.69

**Table 6:** Recall, precision, F-measure, and overall accuracy for each dataset in AlexNet and GoogLeNet.

	CNN architecture	Dataset											
		0.1K	0.5K	1K	2K	3K	4K	5K	6K	7K	8K	9K	10K
Recall	AlexNet	0.39	0.77	0.84	0.82	0.83	0.85	0.81	0.84	0.83	0.84	0.81	0.83
	GoogLeNet	0.34	0.72	0.78	0.79	0.82	0.82	0.75	0.79	0.81	0.75	0.81	0.83

Precision	AlexNet	0.43	0.79	0.85	0.84	0.84	0.86	0.82	0.85	0.86	0.85	0.84	0.83
	GoogLeNet	0.33	0.80	0.81	0.80	0.82	0.83	0.78	0.81	0.83	0.78	0.82	0.84

F-measure	AlexNet	0.32	0.77	0.83	0.81	0.83	0.85	0.80	0.84	0.83	0.84	0.82	0.83
	GoogLeNet	0.30	0.71	0.78	0.79	0.81	0.82	0.74	0.79	0.81	0.74	0.80	0.83

Overall accuracy	AlexNet	0.39	0.77	0.84	0.82	0.83	0.85	0.81	0.84	0.83	0.84	0.81	0.83
	GoogLeNet	0.34	0.72	0.78	0.79	0.82	0.82	0.75	0.79	0.81	0.75	0.81	0.83

## Data Availability

The author has no permission for providing the created models and datasets currently. In order to provide data to a third-party organization in any way, after acquiring consent from the patients and preparing an experiment plan specifying the provision of providing data to the outside, it is necessary to obtain the approval of the ethics committee of the Hokkaido University Hospital. Unfortunately, the author cannot make the data publicly available in the prescribed form.
